# Sick leave patterns in common musculoskeletal disorders – a study of doctor prescribed sick leave

**DOI:** 10.1186/1471-2474-15-176

**Published:** 2014-05-24

**Authors:** Jenny Hubertsson, Martin Englund, Ulf Hallgårde, Ulrik Lidwall, Sofia Löfvendahl, Ingemar F Petersson

**Affiliations:** 1 Department of Orthopedics, Clinical Sciences Lund, Lund University, Lund, Sweden; 2 Epidemiology and Register Centre South, Skåne University Hospital, Medicon Village AB, SE-223 81 Lund, Sweden; 3 Clinical Epidemiology Research & Training Unit, Boston University School of Medicine, Boston, MA, USA; 4 Department for Analysis and Forecast, Statistical Analysis Unit, Swedish Social Insurance Agency, Stockholm, Sweden; 5 Department of Clinical Neuroscience, Division of Insurance Medicine, Karolinska Institutet, Stockholm, Sweden

**Keywords:** Sick leave, Musculoskeletal, Duration of sick leave, Diagnosis, Back pain, Osteoarthritis, Rheumatoid arthritis, Myalgia

## Abstract

**Background:**

Comparative data on sick leave within musculoskeletal disorders (MSDs) is limited. Our objective was to give a descriptive overview of sick leave patterns in different MSDs.

**Methods:**

Using electronic medical records, we collected information on dates and diagnostic codes for all available sick leave certificates, during 2 years (2009–2010), in the North Western part of the Skåne region in Sweden (22 public primary health care centres and two general hospitals). Using the International Classification of Diseases (ICD) 10 codes on the certificates we studied duration, age and sex distribution and recurrent periods of sick leave for six strategically chosen MSDs; low back pain (M54) disc disorders (M51), knee osteoarthritis (M17) hip osteoarthritis (M16) rheumatoid arthritis (M05-M06) and myalgia (M79).

**Results:**

All together 20 251 sick leave periods were issued for 16 673 individuals 16–64 years of age (53% women). Out of the selected disorders, low back pain and myalgia had the shortest sick leave periods, with a mean of 26 and 27 days, respectively, while disc disorders and rheumatoid arthritis had the longest periods with a mean of 150 and 147 days. For low back pain and myalgia 27% and 26% of all sick leave was short (8–14 days) and only 11% and 13%, were long (≥90 days). For the other selected MSDs, less than 5% of the periods were short. For disc disorders, hip osteoarthritis and rheumatoid arthritis, more than 60% of the periods were long (p > 0.001). For back disorders and myalgia most periods were issued in the age groups between 40–49, with similar patterns for women and men. Osteoarthritis and rheumatoid arthritis had most periods in the age groups of 50–64, and patterns for women and men differed. Low back pain, rheumatoid arthritis and myalgia had the greatest share of recurrent sick leave (31%, 34% and 32% respectively).

**Conclusion:**

Duration, age and sex distribution and numbers of recurrent sick leave varies considerably between different MSDs. This underscores the importance of using specified diagnosis, in sick leave research as well as in planning of treatment and rehabilitation and evaluation of prognosis.

## Background

Musculoskeletal disorders (MSDs) are the most common causes of severe long-term pain and physical disability and have a major impact on society [1]. In the Global Burden of Disease Study, from 2010 MSDs were found to cause 21% of all years lived with disability [2]. They were also one of the most common reasons for work disability and sick leave [1]. Sick leave is an important public health problem with both social, economic and health related consequences for the individual as well as social and economic consequences for society [3,4].

There have been several studies on work disability and sick leave in different subgroups of MSDs. Even if different MSDs are often overlapping the group does nonetheless include a wide spectrum of disorders with different disease specific characteristics [1,5]. Low back pain has been reported to cause high amounts of work disability for the overall population, mainly due to its high prevalence [6], while studies of more chronic disorders, like rheumatoid arthritis, instead reports more long lasting work disability in those affected [7,8].

However it is often problematic to compare results from different studies. There are large differences in definitions and terms used and in study design and study measures. Also, differences in social security systems hamper international comparisons [6,7,9].

Furthermore studies of sick leave from Sweden are often based on data from the Swedish Social Insurance Agency (SSIA). For employees, periods of sick leave shorter than 15 days is not registered by the SSIA, and hence not included. As different disorders have different characteristics this might affect studies of the subgroups differently.

We found a need for comparable, basic, descriptive data of sick leave for different MSDs. For future studies we also wanted an estimate of the share of sick leave in different subgroups being sick leave 8–14 days, and hence doctor prescribed sick leave not captured by the SSIA.

The main purpose of this study was to give a descriptive overview of sick leave patterns in different diseases within the group of MSDs, including all doctor prescribed sick leave. The aim was to get comparable estimates of duration, age and sex distribution and patterns of recurrent sick leave for the different subgroups.

## Methods

### Setting and data sources

In Sweden, if you cannot work due to illness or injury you need a certificate from a medical doctor stating the medical reason for a sick leave. This study was based on electronic sick leave certificates collected from the North Western part of the Skåne region in Sweden during the period from January 1st, 2009 until December 31st, 2010. The region has 22 public primary health care units and two general hospitals (in the city/town of Helsingborg and Ängelholm, respectively). Using the electronic medical records, we collected information on dates and diagnostic codes for all available sick leave certificates issued from these units during the study time frame. Certificates starting before but ending within the study time frame and certificates starting within but ending after the study time frame were also included. Sick leave certificates from private primary health care centres or from private corporate medical doctors were not available for inclusion (about 17% of all medical doctor health care visits are estimated to be in private care [10]).

In Sweden, if you are entitled to sickness benefit this is administered by the Swedish Social Incurance Agency (SSIA) and information is registered in the SSIAs registers. For each sick leave period captured trough the medical records, data was therefore linked to data from the SSIA. This was done to capture the full duration of the sick leave periods starting before or ending after the study time frame. It was also done to capture those sick leaves that might have been extended by a private health care provider or at a unit outside the North Western part of the region. Information on diagnostic codes was used from the medical records.

For employed persons the employer will pay the sick pay for the first 14 days. Thus sick leave shorter than 15 days for employees is not captured by the SSIA register. From day 8 this sick leave is however captured by the medical records. Also persons who have not worked in Sweden for the amount of time required, typically 6 months (for example immigrants or young people), or who are supported by social welfare is not entitled to sickness benefit and thus not captured in the register. Therefor for periods not encountered in the SSIAs register only information from the medical records was used.

### Study sample

We required subjects to be aged 18–64 years at the start of the sick leave period as few enter the labour market before 12 years of schooling and the general age of retirement in Sweden is 65 years of age.

### Variables

Sick leave – We defined a sick leave period as a period with a sick leave certificate issued from a medical doctor. In Sweden all sick leaves exceeding 7 days require a sick leave certificate from a medical doctor. In this study only sick leaves with an issued medical certificate is included. This means all sick leaves have at least 7 days of sick leave. However for the included periods the duration of the period is calculated from the first day of sick leave i.e. from day 1.

The exposure variable was disease. As an indicator of disease we have used the International Classification of Diseases (ICD) 10 codes set as the main reason for sick leave by the issuing medical doctor on the initial certificate of the period. The certificate is preceded by a medical examination and is used as the formal basis for the SSIAs decision on compensation. The ICD 10 code should be followed by a description of the patient’s functional and activity limitations and the correctness of the certificates are examined by the administrators at SSIA.

We strategically chose six subcategories, or groups, of MSDs; two back disorders; low back pain (M54) and disc disorders (M51), two types of osteoarthritis, knee osteoarthritis (M17) and hip osteoarthritis (M16) and two disorders with more widespread joint or soft tissue problems respectively; rheumatoid arthritis (M05-M06) and myalgia (M79). The six subcategories were chosen because they represent a broad variety of disorders within the group of MSDs, stretching from acute to more chronic and from specific to less specific disorders, and were relevant to sick leave research.

The outcome variable was days per sick leave period. Days of sick leave was calculated as the number of calendar days of each separate sick leave period. To determine the number of days of a sick leave period all sick leave certificates issued for the same individual with overlapping or connecting dates were paired into one period of sick leave.

In Sweden you can be on sick leave full time or part time of the day. However, as the focus of this study was duration of sick leave, we used only “full” days (e.g., one sick leave day is one real calendar day whether it is with full or part time sick leave). Information on disability pension was not included in this study.

For each individual with a sick leave period due to any of the given diagnostic codes, we also calculated recurrent sick leave periods during the study time frame.

### Study measures and statistical methods

We calculated the number of sick leave periods and the number of sick leave days issued for the whole chapter of MSDs and for each of the six disease groups.

First we calculated proportions of sick leave periods being 8–14 days (short), 15–89 days (intermediate) and ≥90 days (long). We tested differences in proportions being short, intermediate, or long sick leave periods using Pearson Chi-Square test and considered a two-tailed p-value <0.05 to be statistically significant. The test was done on a three by six table comparing proportions of periods between the six disease groups.

Second we calculated mean number of days for the duration of sick leave periods with 95% confidence intervals (CI). We calculated mean number of days for each disease group and for age groups. Age groups were set to 18–29, 30–44, 45–54 and 55–64 years of age, using the age of the individual at the start of each sick leave period. The age groups were chosen to reflect differences in the degree of connection to working life.

In this study we used the full duration of the sick leave periods, including those periods starting before or ending after the study time frame. Because of this, and due to the Swedish social insurance system at the time, some sick leave periods are very long (the sick leave period with the most number of sick leave days in this study had 3204 days) and data contains extreme outliers. Furthermore data is positively skewed and the residuals are not normally distributed. To analyse variance in means we therefore used an ANCOVA with log transformed values using the natural logarithm. In a first step we calculated unadjusted means. In a second step numbers for disease group were adjusted for age and numbers for age groups were adjusted for disease group. For calculations on the total sample of men and women both disease groups and age groups was also adjusted for sex. We then back transformed to geometrical means.

Third we compared the age and sex distribution between the groups. Age and sex distribution was tested separately comparing distribution of periods between the sexes and over the four age groups defined above, between each of the six disorders. Pearson Chi-Square test was used to test for significance.

Finally, we calculated number of individuals with a sick leave period due to each diagnosis who also had one or more recurrent cases during the period and the proportion of those that was issued for the same diagnosis or for another diagnosis within the MSD group. Also here Pearson Chi-Square test was used.

All statistical analysis was done using IBM SPSS statistics 20.

### Ethical approval

The study was approved by the Regional Ethical Review Board at Lund University (Dnr 2011/143). Informed consent was obtained by an opt-out procedure where patients were informed by a notification in the leading newspaper of the area, as recommended by the Regional Ethical Review Board.

## Results

### Sample description

All together 20 251 sick leave periods were issued for 16 673 individuals (8 915 women [53%] and 7 758 men [47%]) with a mean (SD) age of 43 (12) years for women and 43 (13) years for men. In total 19 883 of the included periods (98%) had a valid ICD-10 diagnostic code registered.

### Overall sick leave in MSDs

The group of MSDs was the diagnostic chapter with the highest total number of sick leave periods (3 371 periods [17% of all periods], 1 713 for women and 1 658 for men). The sick leave certificates were issued for 3 088 individuals (1569 women [51%] and 1519 men [49%]). Within the group of MSDs 16% of all sick leave periods were 8–14 days, 54% were 15–89 days and 30% were ≥90 days long (Table 1).

**Table 1:** Number of individuals, total number of sick leave periods and number of periods* being 8–14 days, 15–89 days and ≥90 days long, out of all sick leave periods for each diagnosis

**Women**			Number of periods (%**)			
**Disease group**	**ICD 10**	Individuals (%***)	**Total**	**8-14 days**	**15-89 days**	**≥90 days**
Low back pain	M54	386 (47)	404	102 (25)	259 (64)	43 (11)
Disc disorders	M51	103 (45)	103	5 (5)	29 (28)	69 (67)
Knee osteoarthritis	M17	83 (47)	93	3 (3)	42 (45)	48 (52)
Hip osteoarthritis	M16	16 (36)	16	0 (0)	5 (31)	11 (69)
Rheumatoid arthritis	M05-M06	76 (86)	94	2 (2)	31 (33)	61 (65)
Myalgia	M79	146 (53)	153	40 (26)	85 (56)	28 (18)
MSDs all	M00-M99	1569 (51)	1713	256 (15)	906 (53)	551 (32)
**Men**			Number of periods (%**)			
**Disease group**	**ICD 10**	Individuals (%***)	**Total**	**8-14 days**	**15-89 days**	**≥90 days**
Low back pain	M54	441 (53)	472	137 (29)	283 (60)	52 (11)
Disc disorders	M51	124 (55)	127	1 (1)	46 (36)	80 (63)
Knee osteoarthritis	M17	94 (53)	101	2 (2)	44 (44)	55 (54)
Hip osteoarthritis	M16	29 (64)	34	0 (0)	10 (29)	24 (71)
Rheumatoid arthritis	M05-M06	12 (14)	12	0 (0)	5 (42)	7 (58)
Myalgia	M79	128 (47)	132	35 (27)	87 (66)	10 (8)
MSDs all	M00-M99	1519 (49)	1658	274 (16)	910 (55)	473 (28)
**Women and men**			Number of periods (%**)			
**Disease group**	**ICD 10**	**Individuals**	**Total**	**8-14 days**	**15-89 days**	**≥90 days**
Low back pain	M54	827	876	239 (27)	542 (62)	95 (11)
Disc disorders	M51	227	230	6 (3)	75 (33)	149 (65)
Knee osteoarthritis	M17	177	194	5 (3)	86 (44)	103 (53)
Hip osteoarthritis	M16	45	50	0 (0)	15 (30)	35 (70)
Rheumatoid artritis	M05-M06	88	106	2 (2)	36 (34)	68 (64)
Myalgia	M79	274	285	75 (26)	172 (60)	38 (13)
MSDs all	M00-M99	3088	3371	530 (16)	1817 (54)	1024 (30)

*Difference between proportions tested with Pearson Chi-Square, p < 0.001, for men and women separately and for the total sample.

**Percent of the total periods for that specific disease group.

***Percent of individuals being women and men respecitively, out of total number of individuals with a sick leave due to the diagnosis.

### Duration of sick leave in the different disease groups

Duration of sick leave differed between the disease groups. More than 60% of the sick leave periods for disc disorders, hip osteoarthritis and rheumatoid arthritis had duration of 90 days or more. The corresponding proportions for back pain and myalgia were only 11% and 13%, respectively. On the other hand, for back pain and myalgia as much as 27% and 26% of sick leave periods, respectively, were short (8–14 days) while less than 5% of the periods were classified as short for the other selected MSDs (p > 0.001) (Table 1).

Adjusted for age, the mean number of days per sick leave period was 26 days for low back pain and 27 days for myalgia. Disc disorders and rheumatoid arthritis had the longest periods with a mean of 150 and 147 days respectively. For hip and knee osteoarthritis the mean was 81 and 116 days respectively. Numbers differed only slightly from unadjusted numbers (Table 2).

**Table 2:** Mean* number of days duration for sick leave periods with 95% confidence intervals (CI), unadjusted and adjusted

**Women**	**Unadjusted means**		Adjusted means**	
**Disease group (ICD 10)**	**Mean**	**95% CI**	**Mean**	**95% CI**
Low back pain (M54)	26	23-29	26	24-28
Disc disorders (M51)	161	129-201	150	130-173
Knee osteoarthritis (M17)	90	71-114	81	69-95
Hip osteoarthritis (M16)	120	68-211	116	85-158
Rheumatoid arthritis (M05-M06)	154	122-195	147	119-183
Myalgia (M79)	30	25-36	27	24-31
Age category				
18-29	35	27-46	35	27-46
30-44	48	41-56	48	41-56
45-54	47	40-55	48	41-56
55-64	57	46-71	54	44-67
**Men**	**Unadjusted means**		Adjusted means**	
**Disease group (ICD 10)**	**Mean**	**95% CI**	**Mean**	**95% CI**
Low back pain (M54)	25	23-28	25	23-28
Disc disorders (M51)	138	115-166	139	116-167
Knee osteoarthritis (M17)	77	63-95	75	61-93
Hip osteoarthritis (M16)	118	183-169	114	79-163
Rheumatoid arthritis (M05-M06)	157	87-285	152	84-277
Myalgia (M79)	24	20-29	24	20-29
Age category				
18-29	28	22-36	28	22-35
30-44	40	35-46	40	35-46
45-54	39	33-45	39	33-45
55-64	50	42-60	50	41-59
**Women and men**	**Unadjusted means**		Adjusted means***	
**Disease group (ICD 10)**	**Mean**	**95% CI**	**Mean**	**95% CI**
Low back pain (M54)	26	24-28	26	24-28
Disc disorders (M51)	148	128-171	149	130-172
Knee osteoarthritis (M17)	83	71-97	81	69-95
Hip osteoarthritis (M16)	119	88-161	117	86-159
Rheumatoid arthritis (M05-M06)	154	125-191	148	119-183
Myalgia (M79)	27	24-31	27	24-31
Age category				
18-29	31	26-37	31	26-37
30-44	44	39-48	44	40-49
45-54	43	38-48	43	38-48
55-64	53	47-61	52	45-60
Sex				
Women	47	43-52	46	43-51
Men	40	36-43	40	37-44

*Means are back transformed geometrical means from an ANCOVA on the natural logaritm of the sick leave days.

**Numbers for disease group are adjusted for age and numbers for age groups are adjusted for disease group.

***Numbers for disease group are adjusted for age and sex, numbers for age groups are adjusted for disease group and sex and numbers for sex are adjusted for age and disease group.

### Age and sex distribution of sick leave in the different disease groups

**Figure 1:**
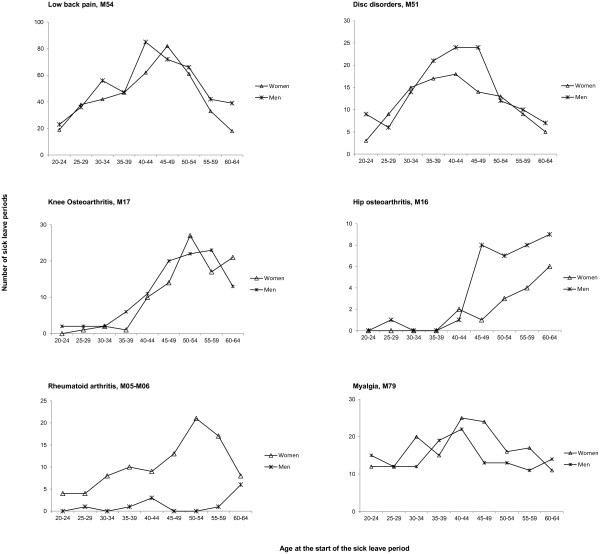
**Age and sex distribution of sick leave periods.** Age and sex distribution of sick leave for each of the six disease groups, presented as total number of sick leave periods in each category.

The distribution of number of sick leave periods, over age categories, and between men and women, was different for the different disease groups. For back disorders (M54 and M51), the total number of sick leave periods was highest in the age groups 40–44 and 45–49, with a similar pattern for women and men. The number of sick leave periods for knee and hip osteoarthritis peaked in the older age groups with a predominance of men in the hip osteoarthritis group. Sick leave for rheumatoid arthritis was predominantly issued for women and peaked in the older age groups. Myalgia had a more even distribution over the age categories with similar patterns for men and women (Figure 1).

### Patterns of recurrent sick leave in the different disease groups

Out of the 3088 individuals with a sick leave issued for an MSD, 25% had more than one sick leave period during the two years. Out of the six studied disease groups, individuals with rheumatoid arthritis had the greatest share of recurrent sick leave periods (34%) despite also having a greater share of long sick leave periods. The other conditions with typically long sick leave periods, e.g., disc disorders and hip osteoarthritis, had less recurrent sick leave (15 and 18% respectively), while typical conditions with short sick leave periods, e.g., back pain and myalgia, had more episodes of recurrent sick leave (31 and 32% respectively) (Table 3).

**Table 3:** Recurrent sick leave, for each disease group and for the total samle of MSDs

**Disease group**	**ICD 10**	**Individuals**	Individuals with recurrent cases (% of total)*	Individuals with recurrent cases M-diagnosis (% of all recurrent cases)**	**Individuals with recurrent cases same diagnosis (% of all recurrent cases)**
Low back pain	M54	827	255 (31)	89 (35)	42 (16)
Disc disorders	M51	227	33 (15)	17 (52)	3 (9)
Knee osteoarthritis	M17	177	42 (24)	30 (71)	14 (33)
Hip osteoarthritis	M16	45	8 (18)	5 (63)	4 (50)
Rhematoid arthritis	M05-M06	88	30 (34)	16 (53)	15 (50)
Myalgia	M79	274	89 (32)	38 (43)	9 (10)
MSDs all	M00-M99	3088	759 (25)	253 (33)	_

*Number of individuals with a sick leave period due to the given diagnosis who also had a recurrent period of sick leave due to any other diagnosis.

**Number of individuals with a sick leave period due to the given diagnosis who also had a recurrent period of sick leave where the sick leave was due to another M-diagnosis.

Individuals with a sick leave due to low back pain, disc disorders and myalgia had a lower share of recurrent periods that were due to the same diagnostic code (16%, 9% and 10% of all recurrent periods respectively) while knee osteoarthritis, hip osteoarthritis and rheumatoid arthritis had a higher share that was due to the same diagnostic code (33%, 50% and 50% respectively) (Table 3).

## Discussion

To our knowledge this is the first study presenting data on differences in sick leave patterns between different MSDs. The study shows that there are clear differences in duration between the different diagnoses within the group. It also shows that there are important differences in the age and sex distribution of the sick leave. This underlines the importance of considering specific diagnosis in sick leave research, not only at the level of diagnostic chapters but at the level of the single diagnoses for which the sick leave is issued.

MSDs are, together with mental and behavioural disorders the main contributors to global years lived with disability. In the Global Burden of Disease Study from 2010, MSDs were found to cause 21% of all years lived with disability. Low back pain and osteoarthritis were two of the main contributors [2]. These years lived with disability entails a great impact on the working lives of the people affected.

Out of the six studied disorders, low back pain had the shortest sick leave periods, with a mean duration of 26 days. Out of all sick leave periods due to low back pain 27% were finished within two weeks. Only 11% were longer than 90 days. This is in line with other research on low back pain [6,11].

Sick leaves labelled as disc disorders however had the longest sick leave periods of the studied disorders, with an adjusted mean of 150 days and 65% of all sick leave periods longer than 90 days (Table 1).

For osteoarthritis, sick leaves for hip osteoarthritis were generally longer than those for knee osteoarthritis. Adjusted for age and sex, the mean duration of a sick leave period was 81 days for knee osteoarthritis and 117 days for hip osteoarthritis. All together 46% of sick leave periods issued for knee osteoarthritis and 71% of sick leaves issued for hip osteoarthritis were 90 days or longer.

Rheumatoid arthritis is the most common form of chronic inflammatory joint disease [12]. The chronicity of the disease is reflected in the high number of long sick leaves, in combination with the largest number of recurrent periods. Adjusted for age and sex the mean duration of a period was 148 days. Of all individuals with an issued sick leave period due to rheumatoid arthritis 34% also had one or more recurrent periods during the study time frame.

Myalgia is maybe the most complex subcategory of the six [13]. The subcategory groups together “other soft tissue disorders not classified elsewhere” and comprises both more regional pain and more widespread pain, including fibromyalgia. Sick leave periods with myalgia in this material were generally short, 26% of the periods were shorter than two weeks and only 14% lasted for 90 days or longer. The adjusted mean duration of a sick leave for myalgia was 27 days.

The age distribution of the sick leave periods differed between the disease groups. Sick leave for back disorders (low back pain and disc disorders) peaked in the age groups 40–44 and 45–49 while sick leave for osteoarthritis followed the typical pattern of osteoarthritis increasing with age [14-16]. Also sick leave for rheumatoid arthritis peaked in the older age groups, as expected [12], while sick leave for myalgia had a more even distribution over the age categories.

Some of the down slope in the older ages might be due to a lesser number at risk in these age groups, as persons with full time disability pension cannot be on sick leave and disability pension is more common in older ages.

The use of the ICD-10 subcategories as we have classified them here can be criticised. Each subcategory in itself contains a number of new subcategories that differ in characteristics. At the same time the subcategories which we have used sometimes overlap. The sick leave certificates on which this study is based also only provides one ICD-10 code for each certificate. Individuals with one MSD tend to have musculoskeletal pain in more than one pain site [17] and other co-morbidities may also influence the experience of the disease, the risk of receiving sick leave and the duration of the sick leave received [18,19].

However, the aim of the study was to examine the variation in sick leave between the groups. The results of the study show that there are great differences in the pattern of sick leave between the subcategories used and that the pattern of sick leave is correspondent to the specific characteristics of the groups.

Disorders with often long sick leave periods had less recurrent periods while those with typically short sick leave periods had more recurrent periods. Part of the reason is probably less time at risk for another sick leave.

About 30% of individuals with a sick leave due to low back pain or myalgia had one or more recurrent periods during the study time frame but only 16% and 10% of those, respectively, were due to the same symptomatic diagnostic code, indicating co-morbid conditions for these patients, and/or that more disease-specific diagnoses are made with time. For all disorders the large number of individuals having additional period(s) of sick leave issued for another MSD is an indicator of the overlap and comorbidity that exists within the group.

Previous studies have argued that the diagnosis of the sick leave certificate is one of the most important factors in determining duration of sick leave [20,21] and return to work [22]. It might be obvious that diagnosis is important for the prognosis - for the simple reason that different diseases have different natural histories and options for treatment, rehabilitation and work adaptations. However despite a medical diagnosis being one of the main prerequisites for the right to sick leave many studies of sick leave do not consider diagnosis [23,24]. And for those that does, including some of those who argue that diagnosis is important, they are often based on main diagnostic groups and all MSDs are treated as one group [20,21,25]. With this article we want to emphasize the importance of also going beyond the level of main diagnostic groups.

By saying this we *do not* suggest that other factors like socioeconomic, work place factors or patient expectations are not important. We simply want to suggest that these factors would also benefit from being studied in relation to the actual diagnosis for which the sick leave is issued. Other studies have reported that the influence of socioeconomic factors as well as the effect of interventions differs between different diagnostic groups [25-27]. To study differences in the association of these factors between subgroups of MSDs is beyond the scope of this article. This is however a subject for further research.

This study has some important limitations. First, the study material does not include certificates from private care givers in the region. Private care givers are more common in primary care than in hospital care. Sick leave that is more commonly issued from hospital care therefore might be overrepresented in the material and eventual differences in age and sex distribution might affect the results. From other studies we know that about 17% of all medical doctor health care visits in the Skåne Region are estimated to be in private care [10].

Second, the study only includes those who have had a sick leave certificate issued. This means that we cannot consider the effect of disability pension (as those with a full time disability pension cannot have sick leave). Disability pension is more common in older ages and in women and this might lead to lower numbers in these groups [28].

Third, the diagnostic code of the sick leave certificates provides the ICD-10 code for diseases or injuries. There is no information on the severity of the disorders. In case of surgeries and sick leave resulting from recovery due to surgery, information is not available. Instead the issuing medical doctor will report the ICD-10 code of the disease that is the underlying reason for the surgery. We therefor do not know if the reason for sick leave is limitations due to the actual disorder or due to recovery after a surgical procedure.

Further, as the aim of the study was to describe duration of sick leave, we have focused on the actual duration of the sick leave, counted as calendar days. This means we are not considering the impact of part time sick leave.

The strength of the study is the access to diagnoses as set by the medical doctor based on a common classification system (ICD-10) and the relatively large material that makes it possible to study single diagnostic groups and diseases. In many earlier studies diagnoses related to sick leave have not been available or have not been utilized.

Furthermore, as the material in this study represents sick leaves issued from medical doctors at the time of the sick leave, this means data is not dependent on response rates leading to selection bias, recall bias or reporting bias.

Using the electronic medical record systems also means that data is not dependent on the rules of the Social insurance system or the approval of the right to sick leave and therefore we capture also those who have never worked or are supported by other types of welfare.

This means that in this study we also capture sick leave for day 8–14, that are not included in the register by the SSIA. As many studies of sick leave from Sweden are based on data from the SSIA, an estimate of the proportions of this short sick leave in the different subgroups can be useful for future studies.

## Conclusion

In conclusion the study shows that duration as well as age and sex distribution of sick leave varies considerably between different diagnostic codes within the group of MSDs. This underlines the importance of considering diagnosis in sick leave research, not only at the level of diagnostic chapters but also for the wide spectrum of different diseases within each chapter. It also emphasises the need of differentiation among MSDs in planning of treatment and rehabilitation and in evaluation of prognosis.

## Abbreviations

MSDs: Musculoskeletal disorders; ICD 10: International classification of diseases 10; SSIA: Swedish Social Insurance Agency.

## Competing interests

The authors declare that they have no competing interests.

## Authors’ contributions

JM organised the collection of data, carried out the analysis and drafted the manuscript. ME participated in the analysis, and supervised the drafting of the manuscript. UH and UL participated in the data collection and the initial planning of the project. SL participated in the analyses of the data. IP supervised the data collection, the planning of the study and the drafting of the manuscript. All authors revised the manuscript critically and approved the final version.

## Pre-publication history

The pre-publication history for this paper can be accessed here:

http://www.biomedcentral.com/1471-2474/15/176/prepub
